# Sarcoidosis With Isolated Osseous Manifestations Mimicking Metastatic Malignancy

**DOI:** 10.7759/cureus.97536

**Published:** 2025-11-23

**Authors:** Ahmed Ismail, Ahmed Saleh, Yanis Boumber

**Affiliations:** 1 Section of Hematology/Oncology, Department of Medicine, O'Neal Comprehensive Cancer Center, University of Alabama at Birmingham, Birmingham, USA; 2 Department of Medicine, Eastern Virginia Medical School, Old Dominion University, Norfolk, USA; 3 Department of Medicine, University of Alabama at Birmingham, Birmingham, USA; 4 Institute of Fundamental Medicine and Biology, Kazan (Volga Region) Federal University, Kazan, RUS

**Keywords:** extrapulmonary, imaging studies, metastatic malignancy, non-caseating granuloma, osseous sarcoidosis

## Abstract

Sarcoidosis is an inflammatory disease of unknown etiology that commonly involves multiple organ systems, with the lungs and lymph nodes most frequently affected. Osseous involvement, although recognized, is considered uncommon and often overlooked due to its nonspecific clinical and radiographic features. Patients may be asymptomatic or present with pain, swelling, and functional limitations of the affected limb. Imaging can reveal findings such as bone lesions that mimic malignancy, including lytic or sclerotic changes. Diagnosis relies on histopathological confirmation of non-caseating granulomas. Treatment is directed at symptom relief and preservation of limb function. Here, we present a rare case of isolated osseous sarcoidosis in a 38-year-old African American female that mimicked metastatic malignancy on imaging and responded dramatically to steroids.

## Introduction

Sarcoidosis is an idiopathic inflammatory disease. It is thought to arise from dysregulated immune activation, leading to persistent non-caseating granuloma formation in various body organs, driven primarily by CD4⁺ T-cell-macrophage interactions. It usually affects the lungs, causing pulmonary infiltrations and bilateral hilar lymphadenopathy on imaging, but it may also involve other extrapulmonary organs. However, osseous sarcoidosis is a rare manifestation of sarcoidosis, reported in approximately 3-13% of sarcoidosis cases. Radiological investigations usually reveal sclerotic or destructive lesions (including joint involvement), cystic and punched-out lesions, and cortical abnormalities. It most commonly affects small bones of the hands and feet. Sometimes, the spine, pelvis, skull, axial skeleton, and ribs are also reported [1]. The diagnosis is confirmed by combining clinical symptoms, such as dyspnea, dry cough, and fatigue, with radiologic findings, such as hilar lymphadenopathy and pulmonary infiltrates, and histologic evidence of non-caseating granulomas on a tissue biopsy [2]. This case highlights a rare presentation of isolated bone sarcoidosis without systemic involvement, emphasizing the importance of considering sarcoidosis in the differential diagnosis of atypical osteolytic lesions.

## Case presentation

Clinical presentation and initial workup

A 38-year-old African American woman presented with worsening lower back pain and MRI findings demonstrating diffuse osseous lesions concerning for metastatic disease. Her symptoms began four years earlier with right hip pain and localized skin retraction, later progressing to involve the lower back and small joints of the hands. She also reported night sweats, nausea, and recurrent focal aware seizures occurring approximately twice weekly, which were controlled with levetiracetam. Her past medical history included iron deficiency microcytic anemia, hypertension, and anxiety, managed with ferrous sulfate, spironolactone, fluoxetine, and lorazepam, respectively. Although her symptoms were initially attributed to pregnancy, they persisted and gradually worsened postpartum.

**Figure 1:**
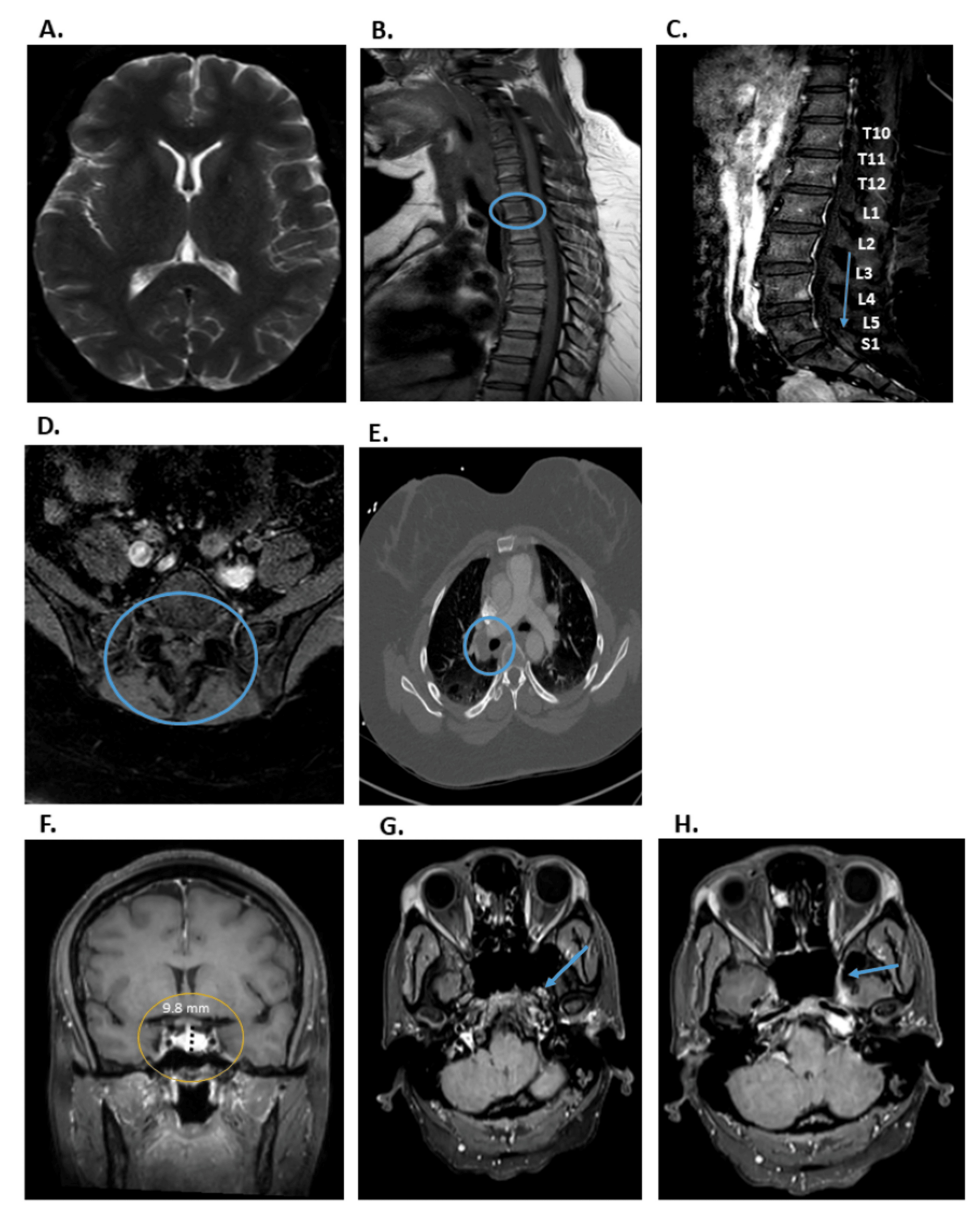
Representative MRI and CT scan images with and without contrast. (A) A representative picture of the initial brain MRI shows no intracranial lesions. (B) A representative picture of the thoracic spine MRI shows a hyper-intense lesion of the T2 vertebral body measuring approximately 9 mm. (C & D) Lumbar spine MRI showing leptomeningeal enhancement in the sacral canal at the level of S1. (E) Thoracic CT with and without contrast showing right hilar adenopathy. (F, G & H) Representative pictures of the repeat brain MRI show mild enlargement of the pituitary gland (9.8 mm) in the F panel and mild thickening and enhancement of the pituitary stalk in the G and H panels.

**Figure 2:**
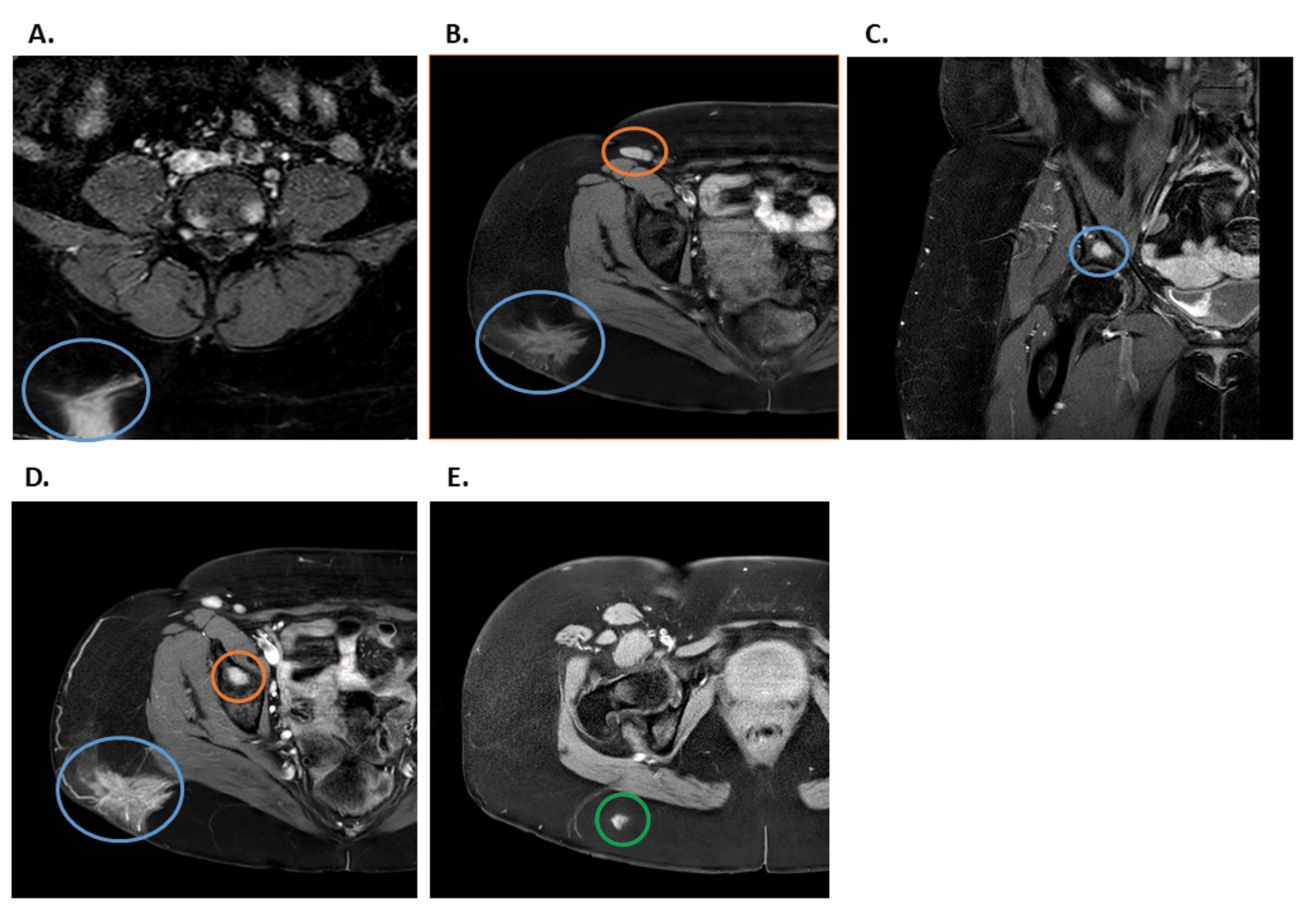
Representative right hip MRI images with and without contrast. The MRI of the right hip and pelvis shows an infiltrative enhancing soft tissue lesion within the subcutaneous right gluteal region (blue circles), followed by another inferior satellite lesion (green circle). Multiple osseous lesions are also visualized within the right hemipelvis (orange circles).

Initial physical exam revealed a firm, retracted, darkened 4-5 cm x 2-3 cm lesion over the right hip with an underlying mass. Her initial lab tests showed elevated C-reactive protein (CRP) and erythrocyte sedimentation rate (ESR) levels (13 mg/L and 77 mm/h, respectively) (Table 1). Her initial imaging studies included CT chest, abdomen, and pelvis with and without contrast scans, which were unrevealing, except for right hilar lymphadenopathy. Brain and spinal MRIs with and without contrast showed no intracranial lesions. Still, they detected sacral leptomeningeal enhancement and multiple spine-enhancing lesions, suggesting bone metastasis and leptomeningeal carcinomatosis. Additionally, an enhancing lesion at the L4 level was found, extending to the skin surface, with suspicion for a primary skin neoplasm or cutaneous metastases (Figure 1). A right hip MRI showed an enhancing soft tissue mass with a satellite lesion and inguinal lymphadenopathy, concerning for sarcoma (Figure 2).

**Table 1:** Comprehensive laboratory and cerebrospinal fluid findings at presentation. WBCs: White Blood cells; Hgb: Hemoglobin; Hct: Hematocrit; RBCs: Red Blood Cells; MCV: Mean Corpuscular Volume; MCH: Mean Corpuscular Hemoglobin; MCHC: Mean Corpuscular Hemoglobin Concentration; RCDW: Red Cell Distribution Width; MPV: Mean Platelet Volume; TIBC: Total Iron Binding Capacity; ESR: Erythrocyte Sidmentation Rate; CRP: C-Reactive Protein; LDH: Lactate Dehydrogenase; ACE: Angiotensin Converting Enzyme; ANA: Anti-nuclear Antibody; C-ANCA: Cytoplasmic Antineutrophil Cytoplasmic Antibodies; P-ANCA: Perinuclear Antineutrophil Cytoplasmic Antibodies; Anti-dsDNA: Anti-double Stranded DNA; Anti-MPO: Anti-Myeloperoxidase Antibody; Anti-PR3: Anti-Proteinase 3; TNC: Total Neutrophil Count

Significant Tests	Results	Reference Range
WBCs	4.5	4-11 * 10^3^/cmm
Hgb	10.8	11.3-15.2 gm/dL
Hct	33%	33%-45%
Platelets	419.8	150-400 * 10^3^/cmm
RBCs	4.26	3.8 - 5.2 * 10^6^/cmm
MCV	78	80-96 fL
MCH	25	27-33 pg
MCHC	32	32-36 gm/dL
RCDW	19.20%	11%-16%
MPV	8	7.5-11.5 fL
Neutrophils	56%	35%-73%
Absolute Neutrophils	2.52	2-7.15 * 10^3^/cmm
Lymphocytes	27%	15%-52%
Absolute Lymphocytes	1.21	1.25-5.77 * 10^3^/cmm
Monocytes	12%	4%-13%
Eosinophils	3	0%-5%
Basophils	2	0%-2%
Iron	35	30-160 mcg/dL
TIBC	368	284-507 mcg/dL
Transferrin Saturation	10%	11%-32%
ESR	77	0-20 mm/hr
CRP	13.01	0-10.9 mg/L
LDH	260	120-240 units/L
ACE	79	9-67 U/L
ANA	<1:80	<1:80
C-ANCA	Positive 1:320	<1:20
P-ANCA	<1:20	<1:20
Anti-dsDNA	<1:10	<1:10
Complement C3	168	>=87.0
Complement C4	34	>=12.9
Anti-MPO	<13	<=20
Anti-PR3	<13	<=20
Vitamin D - 1,25-OH, Total	19	18-72 pg/mL
Vitamin D3 - 1,25-OH	19	18-72 pg/mL
Vitamin D2 - 1,25-OH	<8	<8
Vitamin D 25-OH	15	20-50 ng/mL
CSF Analysis:		
Glucose	51	40-75 mg/dL
Protein	33	18-53 mg/dL
TNC	8	0-5/cmm
RBCs	0	0-1/cmm
Lymphocytes	83%	60-80%
Monocytes	17%	10-30%
Xanthochromia	Absent	Absent
Color	None	None
Clarity	Clear	Clear

Diagnostic evaluation

Given the high probability of malignancy, the decision was to perform multiple biopsies from the detected lesions. A CT-guided gluteal soft-tissue biopsy with simultaneous bone biopsies was performed. The gluteal soft tissue biopsy showed fat necrosis and lymphoplasmacytic infiltrate, and the bone biopsy showed granulomatous inflammation. A mediastinal lymph node biopsy revealed poorly formed granulomas. A bone marrow biopsy, which was done to rule out multiple myeloma, showed no cancer but the presence of non-necrotizing granulomatous inflammation. Additionally, complete CSF studies, including CSF analysis (Table 1) and culture, as well as multiple myeloma workups, were ordered, including serum and urine protein electrophoresis (SPEP and UPEP), blood free light chains (FLC), Immunoglobulins, and lactate dehydrogenase (LDH). However, all of the tests came back negative for infection or malignancy.

With such test results in mind, the possibility of disseminated fungal infection seemed reasonably likely. Further history revealed no personal history of autoimmune diseases, but a history of carpal tunnel surgeries on both wrists. She also had relatives with multiple sclerosis and lupus. The patient lived in a rural area with farm animals nearby, though she did not interact with them and had no pets. Her past employment history was insignificant. Her travel history included travels to Mexico, but no other international destinations. As a teenager, she lived in a household with someone who had tuberculosis and was treated for latent TB. Her physical examination showed a new 3-4 mm erythematous nodule on her right anterior shin, suspicious for erythema nodosum. No other new symptoms or signs were observed. The patient was started on prophylactic amphotericin. However, subsequent microbial tests, including fungal infection tests, returned negative, so amphotericin was discontinued, and further laboratory testing was initiated. At this point, her labs were significant for positive c-ANCA (titer: 1:320), elevated ACE (79 U/L), and low vitamin D 25-OH (15 ng/mL), with negative ANA and dsDNA, and normal complement C3/C4 levels.

**Figure 3:**
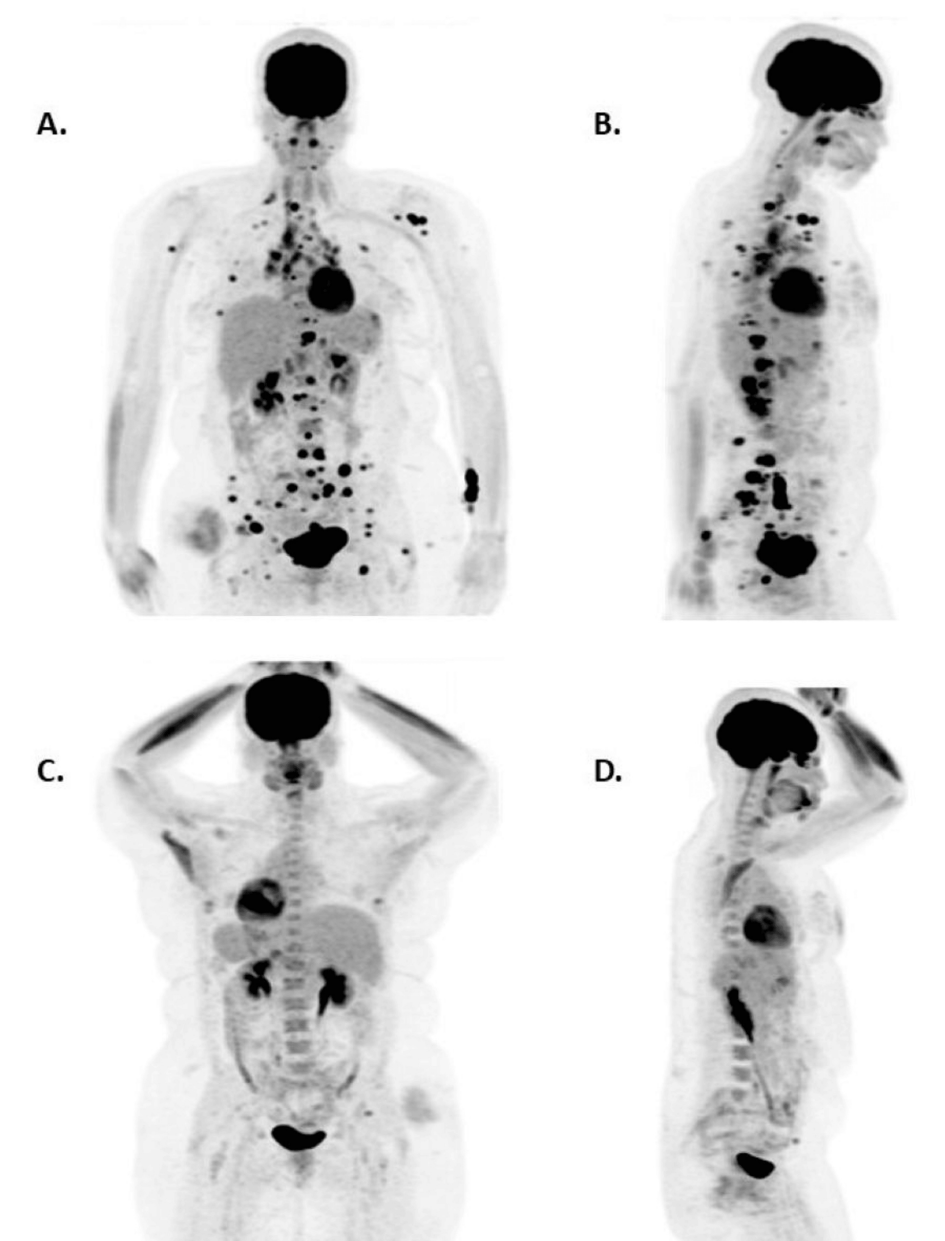
Representative PET scan pre- and post-treatment of sarcoidosis. (A. & B) Pre-treatment PET scan showing extensive metabolically active soft tissue (e.g., lymph nodes on both sides of the diaphragm, lateral hepatic segment, spleen, right buttock) with osseous involvement. (C & D) Post-treatment PET scan showing resolving of the previously seen hypermetabolic foci, consistent with an excellent treatment response.

Furthermore, a repeat brain MRI showed mild thickening and enhancement of the pituitary stalk and mild pituitary gland enlargement, indicating possible neurosarcoidosis or other inflammatory conditions (Figure 1). A PET scan showed generalized hyperactive bone lesions, lymph nodes, pleura, liver, and spleen. Max SUV values ranged from 7.4 (inguinal lymph nodes) to 30.4 (left iliac bone) (Figure 3). A skin biopsy of the new shin lesion showed dermal sclerosis and benign nodular lymphoid aggregates.

Differential diagnosis

Given the patient’s previous clinical history and lab/imaging findings, revised differential diagnoses included granulomatosis with polyangiitis (GPA), metastatic carcinoma, hematopoietic malignancy, ongoing infection, or sarcoidosis. Typical manifestations of GPA were absent, such as recurrent upper respiratory involvement, orbital inflammatory syndrome, lung cavities or multiple lung nodules, cutaneous vasculitis, or glomerulonephritis. Additionally, pachymeningeal involvement is usually more common in GPA than leptomeningeal involvement. Moreover, all the patient's biopsies and tests were negative for malignancy and infection. At that time, disseminated sarcoidosis was a diagnosis of exclusion based on the brain MRI findings, hyperactive lesions on the PET scan, and the granulomas detected on multiple biopsies.

Management and clinical course

The patient was started on systemic treatment for sarcoidosis, including infliximab and prednisone. Infliximab infusions were scheduled every four weeks, and prednisone was started at a dose of 60 mg daily. Prednisone was then tapered by decreasing the dose by 10 mg every two weeks. Additionally, a calcium/vitamin D combination, meloxicam, pantoprazole, and dapsone were added to the regimen. Two months after treatment initiation, ACE levels decreased significantly (from 79 U/L to 20 U/L), and a repeat PET scan showed a dramatic response to treatment, confirming the diagnosis of sarcoidosis (Figure 3). The patient reported feeling much better and that her joint pains had been resolved. Currently, the patient is being treated for disseminated bone sarcoidosis and possible neurosarcoidosis, with a planned 12-month treatment regimen and regular follow-ups. Her condition continues to improve, with no reported drug complications to date.

## Discussion

Sarcoidosis is an inflammatory disease of unknown etiology that affects multiple body systems. However, clinical presentations are usually nonspecific and may include fever, fatigue, myalgia, and arthritis, features also seen in other autoimmune diseases. Although pulmonary sarcoidosis occurs in 90% of cases and is classified into four stages [3] (Table 2), our patient had no pulmonary symptoms and no radiographic lung involvement, making her presentation atypical. Typical presentations of pulmonary sarcoidosis include cough, dyspnea, and chest tightness. However, almost half of the patients with pulmonary sarcoidosis are asymptomatic, especially in the early stages. Stage I shows the best prognosis, with 80% of patients showing spontaneous regression. On the contrary, advanced stages of sarcoidosis only achieve spontaneous regression in one-third of the cases and have a fivefold risk increase of chronic respiratory impairment [4].

**Table 2:** Stages of pulmonary sarcoidosis. Source: Ref. [3], under the Creative Commons CC-BY-NC-ND license.

Stage	Findings
I	Mediastinal/hilar adenopathy (usually bilateral) without pulmonary infiltrates.
II	Mediastinal and hilar adenopathy (usually bilateral) with pulmonary infiltrates.
III	Pulmonary infiltrates without adenopathy (adenopathy has already regressed).
IV	Pulmonary fibrosis with volume loss. No adenopathy.

About 30% of sarcoidosis cases involve extra-pulmonary organs, such as skin, lymph nodes, eye, liver, spleen, heart, nervous system, kidney, parotid gland, nose, larynx, endocrine glands (e.g., thyroid, pituitary, adrenal), bones, skeletal muscles, genitourinary tract, gastrointestinal tract [5-7], while osseous (bone) sarcoidosis is particularly rare, occurring in only 3-13% of patients [8]. Bone involvement commonly occurs in the phalanges of the hands and feet and is usually bilateral. However, the skull, long bones, ribs, pelvis, and axial skeleton may also be affected. While osseous involvement can be asymptomatic, it can sometimes lead to severe disability [9]. In our study, our patient demonstrated multifocal involvement of the pelvis, spine, and small joints of the hands. Imaging techniques are crucial for diagnosis, with radiological investigations revealing sclerotic or destructive lesions (including joint involvement), cystic and punched-out lesions, and cortical abnormalities, which could help diagnose this condition. A biopsy is necessary for differential diagnosis to distinguish these findings from malignancy.

Sarcoidosis has diverse clinical manifestations due to its multi-organ involvement, often mimicking other conditions. It may coexist with several endocrine (e.g., Hashimoto’s thyroiditis, Graves’ disease, type 1 diabetes mellitus, and Addison’s disease) and non-endocrine autoimmune conditions (e.g., systemic lupus erythematosus, rheumatoid arthritis, and Sjögren’s Syndrome) [7]. It may also coexist with malignancies, such as Hodgkin lymphoma, breast cancer, and lung cancer, making the diagnosis more challenging [7]. The final diagnosis, however, is achieved by tissue biopsy revealing non-caseating granulomas with Langhans giant cells [2]. Isolated disseminated osseous sarcoidosis is a challenging entity to diagnose. Its differential diagnosis is broad, including metastatic malignancy (Table 3) [10], vasculitis, fungal infections, and tuberculosis. Careful and dedicated review of systems, along with clinical features, radiologic findings, and definitive tissue biopsy, can help reach the diagnosis. In cases of diagnosis by exclusion, as in our case, a dramatic response to the treatment regimen can confirm the diagnosis and help pave the way for a successful management plan. A limitation of our report is that we were unable to obtain pathology slides, and they were not included in our figures.

**Table 3:** Key differences between bone sarcoidosis and metastatic malignancy to the bone. Source: Ref. [10]

Parameters	Bone Sarcoidosis	Metastatic Malignancy to the Bone
Clinical Presentation	Often part of a systemic disease with other organ involvement (e.g., lungs, skin)	Often associated with a known primary malignancy (e.g., breast, prostate, lung cancer)
	Commonly presents with non-specific symptoms like pain or tenderness in the affected bones	Presents with more severe pain, often worsening at night
	Can be asymptomatic and found incidentally on imaging	Usually causes systemic symptoms like weight loss and fatigue
Radiological Features	Lesions are typically lytic (destructive) or mixed lytic and sclerotic (dense)	Lesions can be purely lytic, purely sclerotic, or mixed
	Lesions are often located in the small bones of the hands and feet (phalanges). The axial skeleton is less common	Commonly affects the axial skeleton (spine, pelvis, ribs) and long bones
	May show a lace-like or honeycomb appearance	Radiographs may show more aggressive and destructive patterns
	PET scans show increased uptake in multiple organs, not just bones	PET or bone scans typically show localized uptake correlating with known cancer sites
Laboratory Findings	May show elevated serum calcium and angiotensin-converting enzyme (ACE) levels	Elevated serum calcium (especially in osteolytic metastases)
	Normal or elevated serum alkaline phosphatase	Elevated serum alkaline phosphatase, especially in cases with extensive bone involvement
	Normal tumor markers	Tumor markers may be elevated depending on the primary malignancy (e.g., PSA for prostate cancer)
Histopathological Examination	Characteristic non-caseating granulomas	No granulomas
	Absence of malignant cells	Presence of malignant cells consistent with the primary tumor
Treatment	Often responds well to corticosteroids or immunosuppressive therapy	Treatment depends on the primary cancer type (chemotherapy, radiation, targeted therapy, immunotherapy)
	Lesions may regress with the treatment of systemic sarcoidosis	Bone lesions may persist or progress despite systemic cancer treatment

## Conclusions

Isolated osseous sarcoidosis is an uncommon presentation that can closely mimic metastatic bone disease, particularly when lesions are diffuse and involve the axial skeleton, as in our patient. Because imaging findings are often nonspecific, distinguishing sarcoidosis from malignancy requires careful clinicoradiologic correlation and, when possible, tissue biopsy.

This case highlights the importance of maintaining a broad differential diagnosis when evaluating multifocal bone lesions and recognizing that sarcoidosis may present without pulmonary or systemic involvement. Early identification prevents unnecessary oncologic interventions and facilitates prompt initiation of appropriate therapy, ultimately improving patient outcomes.
